# Combining Chemotherapeutic Agents, Targeted Therapies, Vaccines and Natural Bioactive Compounds for Mesothelioma: Advances and Perspectives

**DOI:** 10.32604/or.2025.066708

**Published:** 2025-08-28

**Authors:** Raffaele Carrano, Carlotta Zucca, Nicla Cristina, Martina Grande, Eleonora Leti Maggio, Riccardo Bei, Antonio Infante, Chiara Focaccetti, Valeria Lucarini, Loredana Cifaldi, Laura Masuelli, Luciano Mutti, Camilla Palumbo, Monica Benvenuto, Roberto Bei

**Affiliations:** 1 Department of Clinical Sciences and Translational Medicine, University of Rome “Tor Vergata”, Rome, 00133, Italy; 2 Medical School, University of Rome “Tor Vergata”, Rome, 00133, Italy; 3 Department of Life, Health and Health Profession Sciences, Link Campus University, Rome, 00165, Italy; 4 Department of Experimental Medicine, University of Rome “Sapienza”, Rome, 00161, Italy; 5 Department of Applied Sciences and Biotechnology, Università dell’Aquila, L’Aquila, 67100, Italy

**Keywords:** Mesothelioma, chemotherapy, targeted therapy, anticancer vaccines, polyphenols

## Abstract

Mesothelioma is a rare and aggressive cancer with a poor prognosis and limited therapeutic options. Despite recent advances, conventional treatment approaches remain largely ineffective due to late diagnosis, chemoresistance and immunosuppressive tumor microenvironment. This review reports the latest studies on combination therapies for mesothelioma, focusing on the potential of integrating chemotherapeutic agents, molecularly targeted agents, vaccines and natural bioactive compounds such as polyphenols. Clinical and preclinical studies demonstrate that integrating immune-modulating drugs or molecular inhibitors with chemotherapy can improve survival and reduce tumor progression in mesothelioma models and patients. Vaccine-based strategies show potential for inducing host-persistent immune responses when combined with conventional treatments. Moreover, natural compounds such as polyphenols show synergistic effects with chemotherapeutics and targeted agents by modulating several signaling pathways involved in cancer cell growth and progression and by overcoming drug resistance. While several combination strategies are under clinical investigation, further studies are needed to develop more effective and personalized therapeutic approaches that could be translated into standardized treatment protocols.

## 1 Introduction

Mesothelioma is a rare tumor that develops from mesothelial cells that line the serous cavities of the body [1,2]. It frequently arises from the pleura, less frequently from the peritoneal membrane, and, in extremely rare cases, from the tunica vaginalis testis (80%–85%, 10%–15%, and less than 5% of cases, respectively) [1,3]. Given that the vast majority of these tumors are of pleural origin, in this review, the term mesothelioma will be used to indicate pleural neoplasms; otherwise, the different origins will be specified. Mesothelioma primarily affects men older than 60 years of age [4]. Asbestos exposure is the leading risk factor for this tumor, followed by chronic serous inflammation, exposure to various mineral fibers and irradiation [1,5,6]. According to the 2015 WHO classification, mesothelioma is classified into three major histologic subtypes: epithelioid, biphasic, and sarcomatoid. The epithelioid subtype is associated with the best prognosis, whereas the sarcomatoid subtype has the poorest. A high level of epithelial differentiation in biphasic tumors corresponds with longer survival [7].

Mesothelioma is a devastating and highly aggressive malignancy, whose management is further complicated by the late onset of symptoms and by its anatomical position, which makes it difficult to diagnose the neoplasm and treat it with radiotherapy/surgical procedures [4,7]. As a result, this tumor is frequently detected at an advanced stage, with an average life expectancy of 12 to 30 months following diagnosis and a 5-year survival rate of 5%–10% across all tumor stages [4,8]. Until recently, the most effective treatments for mesothelioma have been based on the combination of platinum compounds with folate synthesis inhibitors (e.g., pemetrexed), resulting in a median OS of about 13 months [9]. Chemotherapeutic chemicals can be delivered systemically or by intracavitary administration. Concerning chemotherapy, however, mesothelioma chemoresistance, both intrinsic and acquired, represents an important problem [10]. In this regard, it has been demonstrated that the overexpression of miRNA149 can enhance the expression of the drug efflux membrane protein multidrug resistance protein-1 (MDR-1), also known as P-glycoprotein (P-gp), in mesothelioma cells [10]. Further, mesothelioma cell resistance to both antifolates and platinum compounds has been found to correlate with the expression of the multidrug resistance protein MRP-1 [11,12]. In addition, low levels of the CAAT/enhancer binding protein (C/EBP)-β LIP (LIP), resulting from its proteasome-mediated degradation, have been reported in mesothelioma and associated with cisplatin resistance [13]. Indeed, loss of LIP induces chemoresistance both by increasing P-gp expression and by attenuating endoplasmic reticulum stress-triggered cell death [13]. On the other hand, besides P-gp and MDR proteins expression, mesothelioma chemoresistance appears to rely on multiple mechanisms, still largely undetermined [10].

After the establishment of the pemetrexed-cisplatin combination as a first-line standard of care, several studies aimed at achieving better outcomes for mesothelioma patients have been carried out. The significant results obtained with monoclonal antibodies (mAbs) that target immunological checkpoints (also known as Immune Checkpoint Inhibitors (ICI)) led in 2020 to the approval of the Ipilimumab-Nivolumab combination as a first-line treatment for mesothelioma patients [14,15]. Ipilimumab and Nivolumab promote anti-tumor immunity by targeting, respectively, the cytotoxic T-lymphocyte antigen 4 (CTLA-4) receptor and the programmed cell death protein-1 (PD-1) receptor, which transmit inhibitory signals to T cells. Their approval followed the findings obtained in the CheckMate 743 trial, where the median overall survival (OS) obtained with the ICI combination compared to the platinum-pemetrexed regimen was 18.1 vs. 14.1 months; moreover, the OS rates at 2 years were 41% in the ICI-treated group and 27% in the chemotherapy-treated group [14,15]. However, lately, controversies on the superior benefits offered by ICIs over chemotherapy and concerns on the toxicity associated with ICI therapy have been raised, in particular in patients with mesothelioma of epithelioid histology [16–18]. In any case, the therapeutic options available at present only modestly improve the survival rates for mesothelioma patients.

**Figure 1:**
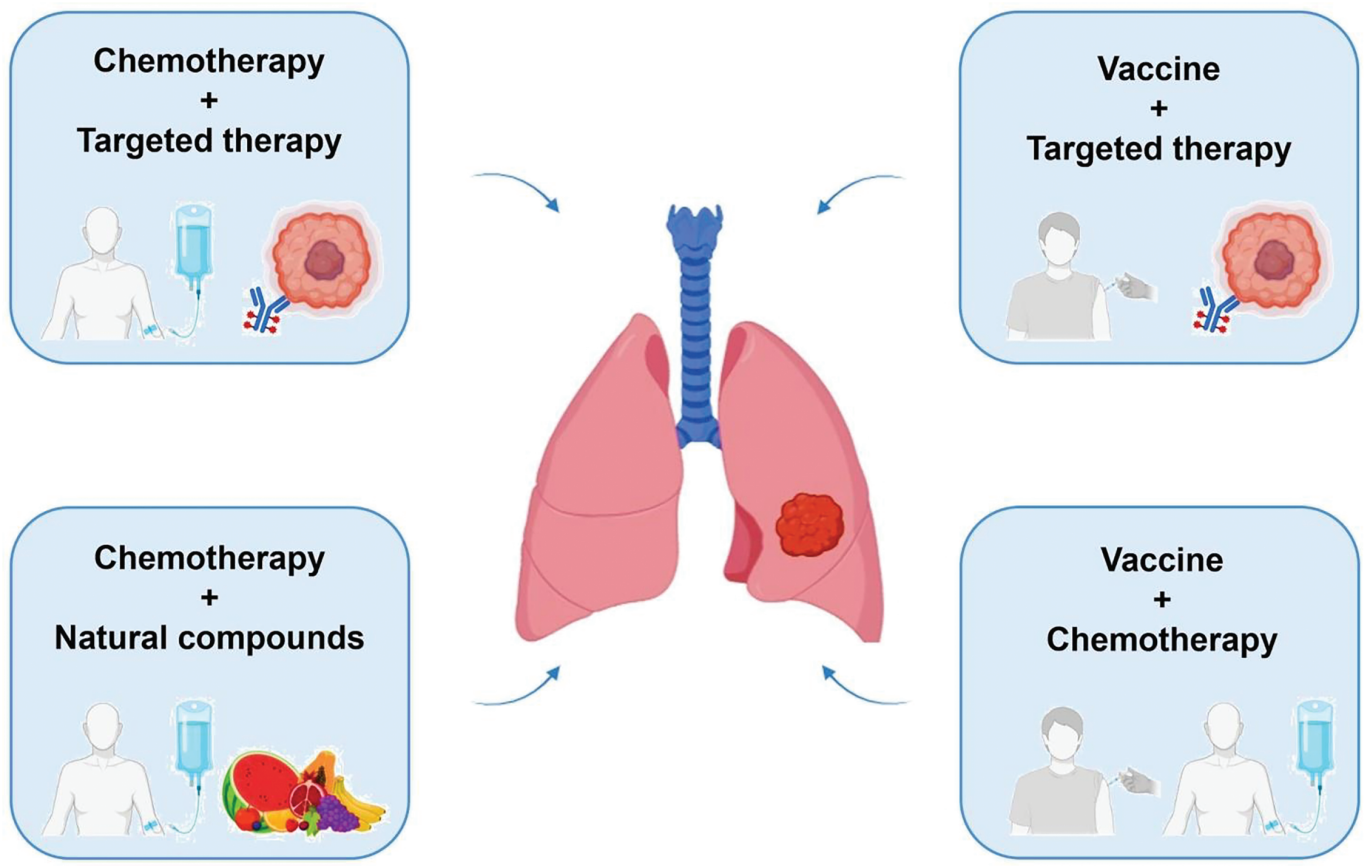
Representation of reviewed combined therapy approaches for mesothelioma. Figure created with BioRender.com

In this review, we report the latest studies on combination therapies for mesothelioma, focusing on the effects of integrating chemotherapeutic agents, molecularly targeted agents, vaccines and natural bioactive compounds such as polyphenols (Fig. 1 and Table 1).

**Table 1:** Combinatorial approaches against mesothelioma

Combinatorial Approach	Preclinical *in Vitro* Model	Preclinical *in Vivo* Model	Clinical Trial	Ref.
**Chemotherapy Plus Targeted Therapy**				
Cisplatin/Oxaliplatin plus RAD001 (Everolimus, mTOR selective inhibitor)	MSTO-211H and non-malignant Met-5A cell lines			[19]
Pemetrexed plus Dasatinib (c-SRC inhibitor)	REN and MSTO-211H cell lines			[20]
Cisplatin plus Entinostat (HDAC inhibitor)	MSTO-211H cell line	CB-17/SCID mice inoculated s.c. with MSTO-211H cells		[21]
Doxorubicin plus anti-mesothelin antibody-functionalized microspheres (APMS-MB) for drug delivery		SCID mice inoculated i.p. with HMESO cells		[22]
Cisplatin/Pemetrexed plus Anti-PD-1 antibody	AB1-HA cell line	C57BL/6 mice inoculated s.c. with AB1-HA cells		[23]
Cisplatin/Pemetrexed plus Durvalumab (anti-PD-L1 antibody)			Multicentre, single-arm, phase II trial (DREAM). 54 patients aged 61–73 years.	[24] Trial ID: ACTRN12616001170415
Platinum compounds/Pemetrexed plus Durvalumab (anti-PD-L1 antibody)			Multicentre open-label, randomised phase III trial (DREAM3R). 480 patients aged ≥18 years.	[25] Trial ID: NCT04334759
Platinum compounds/Pemetrexed plus Pembrolizumab (anti-PD-1 antibody)			Phase III, open-label, randomized controlled trial. 440 patients aged 28–88 years.	[26] Trial ID: NCT02784171
Cisplatin/Pemetrexed plus Bevacizumab (Avastin, anti-VEGF-A antibody)			Randomized, controlled, open-label, phase III trial (MAPS). 448 patients aged 61–70 years.	[27] Trial ID: NCT00651456
Cisplatin/Pemetrexed plus Cediranib (VEGFR/PDGFR inhibitor)			Randomized phase I/II trial (SWOG S0905). 20 (phase I) and 92 (phase II) patients aged 44–85 years.	[28,29] Trial ID: NCT01064648
Cisplatin/Pemetrexed plus Nintedanib (VEGF receptors, PDGF receptors, FGF receptors, Src and Abl inhibitor)			Double blind, randomized, placebo-controlled phase III trial (LUME-Meso). 458 patients aged 58–70 years.	[30] Trial ID: NCT01907100
Carboplatin/Pemetrexed plus Bevacizumab (Avastin, anti-VEGF-A antibody) plus Atezolizumab (anti-PD-L1 antibody)			Randomised, open-label, phase III trial (ETOP 13–18 BEAT-meso). 400 patients aged 42–88 years.	[31] Trial ID: NCT03762018
Cisplatin/Pemetrexed plus Pegargiminase (ADI-PEG20, arginine-degrading enzyme)			Double-blind, randomized, phase II-III trial (ATOMIC-Meso). 249 patients aged 28–86 years.	[32] Trial ID: NCT02709512
**Vaccines Plus Chemotherapy**				
Adenoviral vector expressing IFNα (Ad.IFNα) plus Cisplatin/Gemcitabine		BALB/c mice inoculated s.c. with AB12 cells		[33]
Interferon α-2b (rAd-IFN) plus Celecoxib and Gemcitabine			Open-label, randomized, phase III study. 53 patients aged ≥18 years.	[34] Trial ID: NCT03710876
ONCOS-102 (engineered oncolytic adenovirus encoding for GM-CSF) plus Platinum compounds/Pemetrexed	JL-1, MSTO-211H and NCI-H226 cell lines	BALB/c nude mice inoculated s.c. with NCI-H226 cells		[35]
ONCOS-102 (engineered oncolytic adenovirus encoding for GM-CSF) plus Platinum compounds/Pemetrexed			Open-label, randomised, phase II study. 31 patients aged 36–80 years.	[36] Trial ID: NCT0287966
TroVax (attenuated vaccinia virus encoding for 5T4 TAA) plus Cisplatin/Pemetrexed			Open-label, single-arm, phase II study (SKOPOS). 23 patients aged 61–70 years.	[37] Trial ID: NCT01569919
CRS-207 (attenuated, engineered *Listeria monocytogenes* expressing mesothelin) plus Cisplatin/Pemetrexed			Open-label, phase Ib study. 35 patients aged 51–82 years.	[38] Trial ID: NCT01675765
WT1/DC vaccination plus Cisplatin/Pemetrexed			Multicenter, single-arm, phase I/II study (MESODEC). 28 patients aged ≥18 years.	[39] Trial ID: NCT02649829
WT1/DC vaccination plus Aterolizumab (anti-PD-L1 antibody) plus Cisplatin/Pemetrexed			Multicenter, single-arm, phase I/II study (Immuno-MESODEC). 15 patients aged ≥18 years.	[39] Trial ID: NCT05765084
Autologous DCs loaded with allogeneic mesothelioma cell lysate (MesoPher) after Cytoreductive surgery and Hyperthermic intraperitoneal chemotherapy (CRS-HIPEC)			Open-label, single-arm, phase II study (MESOPEC). 18 patients with peritoneal mesothelioma aged 30–75 years	[40,41]
**Vaccines Plus Targeted Therapy**				
AdV5/3-D24-ICOSL-CD40L (oncolytic adenovirus engineered to express co-stimulatory molecules) plus Pembrolizumab (anti-PD-1 antibody)	H226, MSTO-211H and Mero-82 cell lines	Humanized NGS mice inoculated s.c. with H226 cells		[42]
SS1P (anti-mesothelin immunotoxin) plus Anti-CTLA-4 antibody	AE17M cell line	C57BL/6 mice inoculated i.p. with AE17M cells		[43]
VIC-008 (mesothelin-binding, immune-activating fusion protein) plus AMD3100 (CXCR4 antagonist)		C57BL/6 mice inoculated i.p. with AE17 or 40L cells		[44]
Mesothelin-targeted CAR-T cells plus Pembrolizumab (anti-PD-1 antibody)			Single-arm, open-label, phase I study. 23 patients aged 53–77 years.	[45] Trial ID: NCT02414269
Galinpepimut-S (GPS, WT1-specific peptide vaccine) plus Nivolumab (anti-PD-1 antibody)			Open-label, phase I study. 10 patients aged 59–80 years.	[46] Trial ID: NCT04040231
UV1 (anti-telomerase vaccine) plus Nivolumab (anti-PD-1 antibody) or Ipilimumab (anti-CTLA-4 antibody)			Randomized, open-label, phase II study (NIPU). 118 patients aged ≥18 years.	[47] Trial ID: NCT04300244
**Polyphenols Plus Chemotherapy or Targeted Therapy**				
Curcumin plus Afatinib (pan-ErbB inhibitor)	H-Meso-1, MM-F1, MM-B1, #40a cell lines	C57BL/6 mice inoculated i.p. with #40a cells		[48]
EF24 (curcumin analog) plus Cisplatin	MSTO-211H and Met-5A cell lines			[19]
Resveratrol plus Clofarabine	MSTO-211H and Met-5A cell lines			[49]
Resveratrol plus Cisplatin	MSTO-211H and H-2452 cell lines			[50]
Quercetin plus Cisplatin	SPC111 and SPC212 cell lines			[51,52]
EGCG Plus Ascorbate plus Gemcitabine	REN cell line			[53]

## 2 Chemotherapy Plus Targeted Therapy Combined Approaches

Chemotherapy is still the most effective treatment against mesothelioma, even if the toxicity of traditional anticancer drugs and the development of chemoresistance greatly diminish its effectiveness. Therefore, more and more studies have been, and still are, carried out to explore the combination of chemotherapy and other therapies. Among the most successful ones so far are the ones that combine chemotherapy with targeted therapy approaches. Tumor-targeted treatments use small molecule inhibitors or mAbs to specifically target proteins, including receptors, growth factors or signal transducers, with the purpose of blocking pathways involved in processes such as cell cycle progression, cell survival, angiogenesis and metastasis formation [54,55]. Further, mAbs can also work by potentiating the host immune response by recruiting effector cells and triggering mechanisms such as antibody dependent cellular cytotoxicity (ADCC), antibody dependent cellular phagocytosis (ADCP) and complement dependent cytotoxicity (CDC), or by precisely delivering chemotherapy drugs to cancer cells while minimizing off-target effects [56–58]. Finally, mAbs can boost anti-cancer immune responses by targeting immune checkpoint inhibitory molecules [59].

The protein kinase mammalian target of rapamycin (mTOR) is involved in the regulation of several processes, including cell proliferation, autophagy and apoptosis, and its aberrant activation in mesothelioma makes it a potential therapeutic target for this tumor [60,61]. In this regard, Onen et al. evaluated whether the association between the mTOR-specific inhibitor RAD001 (Everolimus) and platinum-based chemotherapeutics (cisplatin and oxaliplatin) could enhance the antitumor effects of the latter against a mesothelioma cell line (MSTO-211H) [19]. According to their findings, pre-treatment with RAD001 improved the sensitivity to platinum compounds in the tumor cell line, whereas it protected non-malignant mesothelial cells (Met-5A) from the damaging effects of these drugs. These results suggest that this combined strategy could increase apoptosis induction in tumor cells while reducing drug-induced cytotoxic effects in non-malignant cells.

Monica et al. evaluated the effects of a combined treatment composed of pemetrexed and dasatinib [20]. Pemetrexed, a cornerstone drug in mesothelioma treatment, inhibits the action of thymidylate synthase (TS), a key enzyme involved in the conversion of deoxyuridine monophosphate (dUMP) to deoxythymidine monophosphate (dTMP) [62]. The inhibition of TS causes the accumulation of dUMP and an imbalance of deoxynucleotides, provoking DNA damage. Dasatinib is a small-molecule inhibitor of c-SRC, a protein tyrosine kinase with a key role in cell growth and proliferation [63]. Monica and her group observed that the pre-treatment with Dasatinib improved the sensitivity of tumor cells to pemetrexed in two different mesothelioma cell lines (REN and MSTO). Further, the co-treatment induced apoptosis, modulated the PI3K-Akt-mTOR axis and downregulated TS promoter activity [20]. This latter finding is remarkable when considering that high levels of TS expression have been associated with decreased sensitivity to pemetrexed cytotoxic effects in mesothelioma [20].

Histone deacetylases (HDACs), a class of enzymes with an important role in gene expression regulation, are often mutated or dysregulated in different types of cancer. As a result, HDAC inhibitors have been developed as potential anti-cancer agents [64]. One of these inhibitors, Entinostat, was the focus of a study by Schelch et al., who assessed its efficacy against mesothelioma when combined with cisplatin, both *in vitro* and *in vivo* [21]. Entinostat was found to decrease the deacetylation of Y-box-binding protein 1 (YB-1), a pro-tumorigenic nucleic acid-binding protein upregulated in mesothelioma, causing the reduction of mesothelioma cell proliferation. Moreover, this treatment significantly enhanced cisplatin-induced damage and apoptosis of mesothelioma cells *in vitro*, as well as cisplatin-induced inhibition of tumor growth in severe combined immunodeficiency (SCID) mice carrying subcutaneous (s.c.) xenografts of MSTO-211H mesothelioma cells.

Using a completely different approach, Macura et al. developed porous silica microspheres (Acid-Prepared Mesoporous Silica (APMS)) internally loaded with the chemotherapeutic doxorubicin and functionalized on the surface with anti-mesothelin antibodies for tumor targeting [22]. Indeed, being a cell surface glycoprotein expressed in different cancers, including mesothelioma, but not in the parenchyma of vital organs, mesothelin is presently regarded as an attractive therapeutic target in oncology [65]. The doxorubicin-loaded, mesothelin-targeted APMS (APMS-MB-DOX) were then administered via intraperitoneal (i.p.) injection in SCID mice carrying xenografts of human HMESO mesothelioma cells. These targeted microparticles were reported to exhibit superior antitumor efficacy as compared to i.p. doxorubicin. The specificity of the anti-mesothelin antibodies allowed the APMS to efficiently reach the tumor cells, delivering doxorubicin directly to them while minimizing collateral damage to healthy cells. Therefore, targeted microparticles should be evaluated as a desirable choice for targeted drug administration in mesothelioma patients [22].

The efficacy of a combined approach using chemotherapy plus a PD-1-targeted antibody has been investigated in a study conducted by Otsuka et al. In particular, the authors evaluated the effects of a combination of cisplatin/pemetrexed with an anti-PD-1 antibody in a mouse model consisting of AB1-HA mesothelioma tumors growing s.c. in syngeneic C57BL/6 mice [23]. The antitumor activity of the anti-PD-1 antibody was significantly enhanced by the combination with the two drugs, which resulted in a reduced number of intratumoral myeloid-derived suppressor cells (MDSCs), likely associated with the chemotherapy-induced inhibition of Vascular Endothelial Growth Factor (VEGF) expression and tumor-associated blood vessels formation.

The efficacy of the combination of cisplatin/pemetrexed and the anti-programmed cell death ligand-1 (PD-L1) antibody Durvalumab has been investigated in mesothelioma patients in the multicenter, single-arm, phase II DREAM clinical trial [24]. The PD-L1 ligand, expressed by cancer cells and other cells in the tumor microenvironment, binds and activates the PD-1 inhibitory receptor on T lymphocytes. All the study participants had histologically confirmed mesothelioma that was unsuitable for surgical resection and had not been previously treated with systemic therapy. Patients received up to six cycles of cisplatin/pemetrexed and Durvalumab, followed by Durvalumab alone for another 12 cycles. The primary endpoint was progression-free survival (PFS) at 6 months, which was met by 31 out of 54 patients. A total of 60 serious adverse reactions were reported in 29 patients, five of which were directly correlated to Durvalumab. Five patients died during this study, from causes not attributable to the treatments. The results of this trial suggest that the combined treatment with cisplatin/pemetrexed and Durvalumab holds promising effects against mesothelioma, with manageable adverse reactions. Given the outcomes of the DREAM trial, the study was expanded in the phase III randomized DREAM3R trial, in which the number of patients enrolled was increased to 480 participants, with the aim to improve the statistical significance of the data, and for which no results have been published yet [25].

Among ICIs, Nivolumab (currently approved in combination with Ipilimumab for mesothelioma) and Pembrolizumab are both mAbs that target the PD-1 inhibitory receptor, although with different affinities and with a different epitope recognition pattern [66]. The combination of platinum compounds and pemetrexed plus Pembrolizumab, used and well tolerated in non-small-cell lung cancer, has been recently evaluated with encouraging results in a phase III randomized study in which 440 mesothelioma patients were enrolled [26]. A significant improvement in survival parameters was achieved with the addition of Pembrolizumab to chemotherapy: the OS was 17.3 vs. 16.1 months and the 3-year OS rate was 25% vs. 17%, respectively, in the Pembrolizumab plus chemotherapy vs. the chemotherapy alone arm. Thus, on the whole, increasing evidence supports that the combination of different ICIs and cisplatin/pemetrexed chemotherapy holds promise for the therapy of mesothelioma.

Considering the significant role played by angiogenic growth factors in mesothelioma, several clinical studies focused on the combination of chemotherapy and targeted therapy with angiogenesis inhibitors [67–69]. Worthy of note, in addition to its role in angiogenesis, VEGF has been found to act as a key autocrine growth factor for mesothelioma [68]. In this regard, a moderate therapeutic effect and few grade 3 or 4 toxicities were observed in a phase II study in which mesothelioma patients previously treated with platinum-based chemotherapy were treated with Sorafenib, a multi-kinase inhibitor targeting Raf, Platelet Derived Growth Factor receptor (PDGFR) and VEGF receptor (VEGFR) tyrosine kinases [67]. As for the efficacy of approaches based on the simultaneous administration of chemotherapy and angiogenesis-targeting agents, this has been evaluated in the Mesothelioma Avastin Cisplatin Pemetrexed Study (MAPS), Avastin being the brand name for Bevacizumab, a recombinant humanised monoclonal antibody against VEGF-A [27]. In this randomized, controlled, open-label, phase III trial, where all enrolled patients had been recently diagnosed with mesothelioma and had not previously received chemotherapy, a total of 448 patients were selected. The results demonstrated a significant improvement in OS with the addition of Bevacizumab to traditional cisplatin/pemetrexed treatment (median OS 18.8 and 16.1 months for chemotherapy plus Bevacizumab vs. chemotherapy alone, respectively). Although this combination led to anticipated, yet controllable, adverse effects, it was recognized as one of the most promising treatment approaches for mesothelioma. Indeed, based on the results of this study, the cisplatin/pemetrexed/Bevacizumab regimen has been recommended as a first-line option for patients with unresectable mesothelioma in Europe [70,71]. Besides Bevacizumab, different antiangiogenic targeted drugs have been tested in combination with chemotherapy in mesothelioma patients. In this regard, the VEGFR and PDGFR inhibitor Cediranib has been evaluated in combination with cisplatin/pemetrexed in phase I-II studies. However, said combination demonstrated small survival benefits and a toxicity profile that prevented its further development for mesothelioma [28,29]. Further, the combination of cisplatin/pemetrexed with Nintedanib, a triple angiokinase inhibitor targeting VEGF receptors, PDGF receptors and FGF receptors, also able to inhibit Src and Abl kinases, was studied in the LUME-Meso trial, a double blind, randomized, placebo-controlled phase III trial for chemotherapy-naive patients with advanced mesothelioma [30]. However, the primary endpoint of the study was not met since the addition of Nintedanib to standard chemotherapy did not improve patients’ PFS [30].

The potential of a triple combination approach (i.e., a chemo-antiangiogenic-immuno-therapy) has also recently been explored in the BEAT-meso trial, where the effects of carboplatin/pemetrexed/Bevacizumab and the PD-L1-targeting mAb Atezolizumab have been compared with those of chemotherapy plus Bevacizumab alone on a cohort of 400 patients [31]. A strong rationale behind this investigation relies on the evidence that VEGF can participate in suppressing anti-tumor immunity via multiple mechanisms and that, accordingly, VEGFR inhibitors could synergize with ICIs [72]. The results of the study support further investigations on the role of ICI-based combination therapies for mesothelioma. Indeed, although OS was not significantly different between the two groups of patients, a significantly increased benefit was obtained with the triple combination regimen in terms of PFS and duration of response in patients with non-epithelioid mesothelioma [31].

In the context of combined approaches involving angiogenesis-targeting agents and ICIs, it is worth mentioning a single-arm, phase II study in which the anti-PD-1 antibody Pembrolizumab has been evaluated in combination with the antiangiogenic multikinase inhibitor Lenvatinib in a small cohort of patients with mesothelioma who progressed after standard chemotherapy [73]. Although this approach was found to have a relevant toxicity, it showed promising antitumor activity in terms of both response rate and duration of response. Based on these encouraging results, a second cohort of patients has been recruited; in this cohort, the Pembrolizumab-Lenvatinib combination will be evaluated in patients pretreated with nivolumab plus ipilimumab only (NCT04287829) [73].

About 60% of non-epithelioid mesothelioma cases and 20% of mesothelioma cases with epithelioid histology are arginine-auxotrophic due to the lack or decreased expression of arginine succinate synthetase 1 (ASS1), which catalyzes the rate-limiting step in the biosynthesis of this amino acid [74]. Accordingly, arginine-depleting agents have been evaluated as therapeutic strategies in ASS1-deficient mesothelioma. In particular, the pegylated arginine-degrading enzyme arginine deiminase (ADI-PEG20 or pegargiminase) has shown some survival benefits as a single agent in ASS1-deficient mesothelioma [75]. Based on these results and on the evidence that ADI-PEG20 can sensitize tumors auxotrophic for arginine to pemetrexed effects, the combination of cisplatin/pemetrexed and ADI-PEG20 has been investigated in chemotherapy-naïve patients with unresectable non-epithelioid mesothelioma in the ATOMIC-Meso (ADI-PEG20 Targeting of Malignancies Induces Cytotoxicity-Mesothelioma) trial, whose results have been recently published [32]. A total of 249 participants were enrolled in this randomized, placebo-controlled, phase III study and were treated with ADI-PEG20 plus standard cisplatin/pemetrexed chemotherapy or placebo plus chemotherapy. The OS of patients in the ADI-PEG20/chemotherapy arm was 9.3 months, compared to 7.7 months for those in the placebo/chemotherapy arm, while the PFS were 6.2 and 5.6 months, respectively. The survival rate at 36 months was 11.9% for patients receiving ADI-PEG20/chemotherapy vs. 3.3% for those receiving placebo/chemotherapy. The results provided by this clinical trial thus support the combination of arginine depletion and chemotherapy as a possible strategy for increasing survival outcomes in patients with ASS1-deficient mesothelioma.

Overall, based on the available evidence, the combinations of chemotherapy and targeted agents that appear to provide better results are those involving the use of ICIs or angiogenesis-targeting agents. In this regard, a comparative analysis of different combination regimens has been the subject of recent meta-analysis studies [76,77].

## 3 Vaccines Plus Chemotherapy Combined Approaches

In recent decades, numerous studies have been done in order to increase the host immune response against cancer cells by using therapeutic vaccines [78]. In order to effectively educate the immune system to recognize cancer cells, specific antigens must be employed, which are categorized into Tumor Specific Antigens or neoantigens (TSAs) and Tumor Associated Antigens (TAAs) [78]. TSAs are mutated proteins that are expressed exclusively by neoplastic cells, whereas TAAs are non-mutated self-proteins overexpressed in cancerous cells, such as the Human Epidermal Growth Factor Receptor 2 (HER2), Mucin-1 (MUC-1), human Telomerase Reverse Transcriptase (hTERT) [79–81]. The main TAAs in mesothelioma are mesothelin and the Wilms Tumor-1 (WT1) protein [82]. Mesothelin is expressed in the majority of epithelioid mesothelioma cases, whereas WT1 is expressed in both epithelioid (70%–100%) and sarcomatoid (10%–45%) mesothelioma [65,82–84]. Lately, the trophoblast glycoprotein (5T4), an oncofetal protein with low expression in normal tissues, has been included among mesothelioma TAAs and found to be equally expressed in all histological subtypes [85,86]. As for TSAs, mesothelioma has been traditionally regarded as a tumor with a low mutational burden and thereby predicted to have a limited neoantigen expression [83]. However, a growing body of evidence supports the existence of candidate neoantigens in mesothelioma, which, if validated, may open the way for personalized cancer immunotherapy strategies in the future [87,88].

Actually, the term “cancer vaccines” refers to a broad variety of therapeutic strategies aimed at mobilizing immune responses for the identification and eradication of neoplastic cells. These approaches are designed to stimulate tumor-specific cytotoxic T lymphocyte (CTL) activity or enhance antigen presentation, ultimately promoting immune-mediated tumor clearance. Vaccination strategies include the administration of antigens in the form of synthetic peptides or full-length proteins, antigen-encoding nucleic acids, antigen-loaded dendritic cells (DCs), as well as oncolytic viruses or viruses modified to express tumor antigens or immunomodulatory molecules [89].

Vaccine-based approaches represent an attractive strategy to fight mesothelioma, and some evidence of efficacy has been reported in clinical studies in which therapeutic vaccines have been used as monotherapy [90–92]. On the other hand, the immunosuppressive microenvironment of this tumor is regarded as a main factor able to limit the effectiveness of vaccination therapies. Therefore, many studies are presently focused on the combination of therapeutic vaccines with chemotherapy or ICIs [90–93]. In fact, chemotherapy not only reduces tumor burden but can also modulate the tumor microenvironment by increasing antigen availability through immunogenic cell death, depleting immunosuppressive regulatory T cells, and enhancing antigen presentation. Thus, chemotherapy may amplify the efficacy of cancer vaccines, and *vice versa* [93,94]. In this context, the definition of the immunomodulatory effect of different chemotherapeutic drugs and modes of drug administration is paramount to guide the rational design of strategies based on immunotherapy-chemotherapy combinations [94,95].

Fridlender et al. investigated whether chemotherapy could enhance the effects of an *in situ* vaccination approach based on the adenoviral vector (Ad)-mediated delivery of genes encoding for immunostimulatory molecules directly in the tumor microenvironment [33]. In this study, BALB/c mice bearing syngeneic AB12 mesothelioma tumors were intratumorally injected with Ad-expressing IFN-α (Ad.IFN-α), followed by weekly treatments with a combination of cisplatin and gemcitabine. The authors expected that the vaccination could prime an initial strong antitumor immune response, which next could be boosted by the chemotherapy-mediated killing of tumor cells and the ensuing release of immunostimulatory tumor antigens. Indeed, while both Ad.IFN-α and chemotherapy individually inhibited tumor growth (with tumor shrinkage in 5/15 and 2/15 animals, respectively), their combination had a markedly increased antitumor efficacy (complete tumor regression in 13/15 animals). In addition, in this study chemotherapy enhanced the effects of immunotherapy via multiple pathways, including by increasing the number of systemic antitumor cytotoxic T-lymphocytes [33]. The efficacy and safety of Ad-mediated intrapleural delivery of Interferon α-2b (rAd-IFN) in combination with celecoxib and gemcitabine in patients with mesothelioma (epithelioid or biphasic histology), who have failed previous treatments, is currently being evaluated in a phase III, open-label, randomized study. The primary goal of the study is to compare the OS in patients receiving rAd-IFN with celecoxib and gemcitabine vs. those receiving celecoxib and gemcitabine alone [34]. Of note, cyclooxygenase-2 (COX-2) inhibition by celecoxib has been previously shown to improve immunotherapy efficacy in preclinical studies, providing a rationale for testing their combination in the clinical setting [96,97].

A different strategy for *in situ* cancer vaccination is the one based on oncolytic viruses. These are naturally occurring or recombinant viruses that selectively infect and lyse cancer cells and thus exert an anticancer effect both through their direct cytotoxic activity and by altering the tumor microenvironment, recruiting immune cells and enhancing the overall antitumor immune response [89,98]. ONCOS-102 is an oncolytic adenovirus genetically modified for enhanced gene delivery to cancer cells and encoding the Granulocyte-Macrophage Colony-Stimulating Factor (GM-CSF), which is a potent inducer of antitumor immunity. In a preclinical study by Kuryk et al., the efficacy of the standard of care chemotherapy (platinum compounds/pemetrexed) combined with ONCOS-102 was evaluated in mesothelioma *in vitro* and *in vivo*. These authors observed that ONCOS-102 induces immunogenic cell death in mesothelioma cell lines *in vitro* and has anti-tumor effects in a mouse xenograft model of treatment-refractory mesothelioma. In fact, in the murine mesothelioma model, ONCOS-102 inhibited tumor growth, whereas chemotherapy alone did not. More interestingly, combining ONCOS-102 with chemotherapy regimens resulted in synergistic anti-tumor effects [35]. These findings have supported the clinical testing of ONCOS-102 in combination with first-line chemotherapy in patients with mesothelioma. ONCOS-102 was first clinically evaluated in a phase I study performed in 12 patients with late-stage cancers (including 2 cases of mesothelioma) refractory to available treatments. Patients were repeatedly treated by intratumoral injection of ONCOS-102 plus daily low-dose oral cyclophosphamide. The study proved the safety and immune-stimulating capabilities of the vaccination, and signals of clinical efficacy were reported [99]. Next, a randomized phase II study was performed aimed at evaluating whether the combination of ONCOS-102 with standard chemotherapy could improve treatment outcomes in mesothelioma patients [36]. In this study, 31 patients with unresectable mesothelioma, previously treated or not with chemotherapy, were enrolled, who received repeated intratumoral injections of ONCOS-102 and/or chemotherapy with platinum compounds plus pemetrexed. The combined vaccination-chemotherapy approach was feasible and well tolerated, and a survival advantage over chemotherapy alone was possibly obtained in chemotherapy-naïve patients. However, the small number of patients enrolled precluded the possibility of drawing definitive conclusions in this regard [36,100]. Interestingly, in the ONCOS-102-treated group, survival at 18 months appeared to correlate with early immune activation, evaluated in terms of elevation of T-cell infiltration and expression of immune response-related genes in tumor biopsies collected at day 36. These observations support future investigations on the efficacy of ONCOS-102 in combination with ICIs in patients with mesothelioma.

A vaccine against the 5T4 TAA has been developed and tested in mesothelioma in combination with chemotherapy. This “TroVax” vaccine, consisting of a highly attenuated vaccinia virus containing the gene encoding for the 5T4 trophoblast glycoprotein, has been shown to be well tolerated and effective in inducing 5T4-specific immune responses in patients with different types of cancer [37]. The efficacy of TroVax in combination with cisplatin/pemetrexed has been evaluated in the SKOPOS trial: an open-label, single-arm phase II study including patients with locally advanced or metastatic mesothelioma [37]. Eligible patients received up to 9 intramuscular injections of TroVax, beginning two weeks before chemotherapy and continuing at regular intervals for 24 weeks. Among the 27 patients enrolled, 23 (85%) received at least three doses of TroVax and one cycle of chemotherapy. A humoral or cellular immune response to 5T4 was observed in 22 out of 23 patients (95.6%), achieving the primary goal of the study. Disease control occurred in 87% of patients, with a median PFS of 6.8 months and a median OS of 10.9 months. Treatment-related adverse effects were comparable to those typically seen in patients receiving chemotherapy alone. The strong immunological activity and adequate tolerability of the TroVax-chemotherapy combination support a future phase III investigation in mesothelioma patients [37].

A further vaccination strategy clinically assessed in mesothelioma patients is based on the CRS-207 vaccine, an attenuated *Listeria monocytogenes* engineered to express the TAA mesothelin. The efficacy of CRS-207 was assessed in a phase Ib trial in combination with pemetrexed and cisplatin, achieving a median PFS and OS of 7.5 and 14.7 months, respectively. Interestingly, tumor size decreased following CRS-207 infusion prior to chemotherapy in 11 out of 35 patients (31%). No unexpected treatment-related major adverse events or deaths were reported. Analysis of tumor biopsies pre- and post-CRS-207 treatment revealed potential T cell reinvigoration and proliferation, increased infiltration of dendritic and natural killer cells, increased CD8/Treg ratio, and a shift from immunosuppressive M2-like to proinflammatory M1-like macrophages after CRS-207 administration. Overall, the combination of CRS-207 with chemotherapy resulted in significant alterations in the tumor microenvironment and objective tumor responses in the majority of the treated patients [38].

A different strategy that has been used in different trials in association with standard first-line chemotherapy is the vaccination with autologous DCs loaded with tumor cell lysates [101,102]. In phase I trials, this approach has demonstrated acceptable safety and some evidence of clinical efficacy [103,104].

In the phase I/II MESODEC trial (estimated study completion in 2025), vaccination with autologous DCs loaded with the mRNA encoding for the WT1 TAA (WT1/DC) has been combined with standard chemotherapy for the first-line treatment of epithelioid mesothelioma. Meanwhile, a multicentre, single-arm, phase I/II trial (immuno-MESODEC) is currently underway in which vaccination with WT1/DC is combined with conventional platinum/pemetrexed-based chemotherapy and with the anti-PD-L1 antibody Atezolizumab in 15 treatment-naïve patients with inoperable epithelioid mesothelioma [39]. Although the study has a small sample size and lacks a control arm, an indirect comparison could be made with the findings of the MESODEC trial, and the results may guide the future development of DC-based therapies for this hard-to-treat cancer.

In patients with peritoneal mesothelioma eligible for cytoreductive surgery (CRS) and hyperthermic intraperitoneal chemotherapy (HIPEC), this approach has resulted in a median survival of 53 months and a 5-year survival rate of 47%. However, recurrence rates remain high [105]. The feasibility of using autologous DCs loaded with an allogeneic lysate obtained from mesothelioma cell lines (MesoPher) following CRS-HIPEC has been evaluated in the phase II trial MESOPEC in patients with peritoneal mesothelioma of epithelioid histology [40]. The treatment was reported to be feasible and safe, and to induce a positive immune modulatory effect on lymphoid cells. In particular, it was associated with increased proliferation of circulating natural killer cells and CD4^+^ T helper (Th) cells. Following therapy, co-stimulatory molecules, such as ICOS, HLA-DR, and CD28, increased primarily on memory or developing Th cells. Moreover, an increase in CD8^+^ terminally differentiated effector memory cells was positively correlated with PFS. These findings support future combination therapy options for peritoneal mesothelioma [41].

More recently, the efficacy of MesoPher DCs has been investigated in patients with pleural mesothelioma in the DENIM trial: an open-label, randomized, phase II/III study comparing the efficacy of MesoPher plus best supportive care vs. best supportive care alone, as a maintenance therapy after standard chemotherapy. Although the MesoPher treatment did not improve OS compared with best supportive care alone, it had a good safety profile and was associated with signs of immunological activation. Based on this evidence, it is advised to carry on further studies to evaluate whether the combination with MesoPher might increase the efficacy of ICIs in mesothelioma patients [106].

Ultimately, due to the immunosuppressive features of mesothelioma, the clinical translation of therapeutic vaccines for this tumor is geared toward combining them with agents capable of boosting antitumor immunity, such as chemotherapeutic drugs or, as discussed in the next paragraph, immunological checkpoint-targeting agents.

## 4 Vaccines Plus Targeted Therapy Combined Approaches

Interesting approaches recently evaluated against mesothelioma are based on vaccines plus targeted therapy combinations. In this context, a novel therapy based on the combination of the oncolytic adenovirus AdV5/3-D24-ICOSL-CD40L and the anti-PD-1 mAb Pembrolizumab has been tested by Garofalo et al., *in vitro* and in a mesothelioma mouse model. AdV5/3-D24-ICOSL-CD40L is an oncolytic adenovirus engineered to express two potent co-stimulatory molecules aimed at amplifying the antitumor immune response, i.e., the inducible co-stimulator ligand (ICOSL) and CD40 ligand (CD40L, CD154). ICOSL binding to its cognate receptor ICOS expressed on T cells induces cytotoxic T lymphocytes activation, while CD40L binding to the CD40 receptor, expressed on B cells, macrophages and DCs, induces the activation of adaptive immune responses via multiple mechanisms [42,107]. The combination of AdV5/3-D24-ICOSL-CD40L and Pembrolizumab significantly reduced survival in H226, Mero-82 and MSTO-211H human mesothelioma cell lines cultured *in vitro*, with an increase in immunogenic cell death. Moreover, in *in vivo* experiments, performed on mesothelioma xenografts established by subcutaneous injection of H226 cells in humanized NGS mice (human CD34^+^ hematopoietic stem-cell-engrafted mice), the reduction in tumor size achieved with the combined treatment was much higher than that obtained with each of the two agents used individually. Noteworthy, an enhanced presence of activated tumor-infiltrating T lymphocytes was observed in mice treated with the combination therapy. These results support further assessment of AdV5/3-D24-ICOSL-CD40L in combination with ICIs as a novel therapeutic perspective for mesothelioma [42].

Since the discovery of mesothelin, different strategies have been developed to target this TAA [65]. Among these is the recombinant anti-mesothelin immunotoxin SS1P, consisting of an anti-mesothelin antibody fragment fused to a truncated portion of Pseudomonas exotoxin A; SS1P binds, is internalized and induces cell death in mesothelin-expressing cells. However, in different phase I trials on mesothelioma patients, minor responses have been obtained with SS1P alone [108]. Leshem et al. performed a study in a syngeneic mouse model, based on the growth of mesothelin-expressing AE17M mesothelioma tumors in C57BL/6 mice, and demonstrated that SS1P treatment can increase the efficacy of CTLA-4 blockade [43]. In this study, treatment with SS1P induced immunogenic cell death in AE17M cells cultured *in vitro*. Moreover, the intra-tumoral injection of SS1P was shown to enhance the efficacy of an anti-CTLA-4 ICI, administered i.p., in tumor-bearing mice. In fact, tumor size was significantly reduced in 3 out of 8 mice treated with anti-CTLA-4 alone, with complete tumor eradication in two of them, whereas tumor size was reduced in all the mice receiving the combined treatment, with complete eradication in 7 out of 8 of them. Remarkably, following the re-injection of tumor cells, the surviving mice were shown to be protected from tumor regrowth, indicating the development of long-term anti-tumor immunity. According to these findings, the use of intra-tumoral SS1P in combination with anti-CTLA-4 warrants future investigations in mesothelioma patients [43].

A further strategy exploiting mesothelin as a target is the immune-activating fusion protein VIC-008 [109]. VIC-008 is an improved version of a bifunctional fusion protein composed of a mesothelin-binding single-chain antibody variable fragment (scFv) and *Mycobacterium tuberculosis* heat shock protein 70 (MTBHsp70), the latter of which acts as a potent immune adjuvant. This fusion protein has been proven to exert antitumor effects mediated by tumor-specific CD8^+^ T cells in mouse models of mesothelin-expressing cancers, including mesothelioma [44,109]. The efficacy of combining vaccination with VIC-008 and AMD3100 (Plerixafor), a non-peptide antagonist of the chemokine receptor 4 (CXCR4), has been investigated by Li et al. in two syngeneic models of mesothelioma in immunocompetent mice [44]. The rationale behind this combination strategy relates to the involvement of CXCR4 and its ligand CXCL12 in supporting tumor cell growth and angiogenesis, as well as immunosuppression in the tumor microenvironment [110]. In this study, the treatment with VIC-008 was found to increase mesothelin-specific CD8^+^ T cell responses, to promote lymphocytic infiltration in the tumor microenvironment, but also to induce an increased expression of PD-1 in intratumoral CD8^+^ T lymphocytes. On the other hand, AMD3100, alone or combined with VIC-008, was able to suppress PD-1 expression on CD8^+^ T cells and to reduce tumor-infiltrating immunosuppressive Tregs, possibly by mediating their conversion into helper-like cells. Indeed, the two agents in combination demonstrated a synergistic effect, leading to improved tumor growth control and to a significant increase in the mean OS of treated mice.

The efficacy of combined approaches, that integrate immunostimulatory techniques and targeted therapy agents, has also been clinically assessed in mesothelioma patients. In this regard, a first-in-human phase I study, in which a locoregional treatment with autologous, mesothelin-targeted chimeric antigen receptor (CAR) T cells has been combined with the anti-PD-1 Pembrolizumab, has been conducted [45]. In this study, 23 previously treated patients with mesothelin-positive mesothelioma were intrapleurally infused with mesothelin-targeted CAR T cells. After about 20 months since T cell infusion, median OS was 17.7 months and 1-year OS was 74%. Treatment with Pembrolizumab after 4–17 weeks from T cell infusion was performed in 18 of these patients and improved median OS to 23.9 months and 1-year OS to 83%. Based on this study, proving the safety and feasibility of the proposed combination therapy, a phase II trial is underway with the aim of optimizing the efficacy of the combined approach by using a fixed dose of mesothelin-targeted CAR T cells, followed by the initiation of Pembrolizumab 4 weeks after CAR T-cell administration. The estimated study completion is April 2026 [45].

The TAA WT1 has also been exploited as a target for mesothelioma vaccine therapy [80], and the efficacy of a combined approach composed by a WT1-specific peptide vaccine (Galinpepimut-S, GPS) and the anti-PD-1 mAb Nivolumab has recently been evaluated in a phase I trial in mesothelioma patients with WT1-positive tumors [46]. Ten patients with progressive/recurrent mesothelioma previously treated with at least one course of chemotherapy were enrolled in the study. In summary, the study reported that the combination of GPS and Nivolumab was safe and well tolerated, able to induce vaccine-specific T cell responses in 30% of patients but associated with suboptimal objective responses and survival outcomes. Possibly, a better assessment of the efficacy and potential of this approach would require further research on a larger number of patients [46].

Lastly, a randomized phase II trial is underway to compare the efficacy of two different ICIs (the anti-CTLA-4 Ipilimumab and the anti-PD-1 Nivolumab) combined or not with a vaccine targeting telomerase (UV1) [47]. This therapeutic anti-cancer vaccine is known to induce immune responses against the telomerase reverse transcriptase (hTERT), i.e., the catalytic subunit of the telomerase complex, which, by maintaining telomere length in dividing cells, plays an essential role in tumor growth. The study will be conducted in 118 mesothelioma patients who have progressed after standard first-line chemotherapy and will demonstrate whether improved responses to ICI therapy could be obtained as a result of their combination with the UV1 vaccine. Positive findings in this regard may be transferred to the first-line setting.

On the whole, preclinical and early-phase data support the potential of combining tumor-targeted vaccines with checkpoint blockade or immune modulation in mesothelioma, although further optimization of the combined approaches and clinical validation are needed.

## 5 Integrating Polyphenols, Chemotherapy and Targeted Therapy

Polyphenols are a large group of naturally occurring compounds that come from plants and are thus found in foods and beverages, such as cereals, nuts, legumes, olives, tea, coffee, wine, fruits, vegetables, and spices [111]. These natural compounds have been reported to modulate multiple signal transduction pathways involved in carcinogenesis, both *in vitro* and *in vivo*. Indeed, polyphenols have strong modulatory effects on growth factor receptors [including VEGFR, the Epidermal Growth Factor Receptor (EGFR) and Human Epidermal Growth Factor Receptor 2 (HER2/ErbB2), Insulin-like Growth Factor 1-Receptor (IGF1-R)], signal transducers [including Ras/Raf, mTOR, Phosphatidil-Inositol-3-Kinase (PI3K), Bcr-Abl, AMP-activated protein Kinase (AMPK)], transcriptional regulators [such as Nuclear Factor Erythroid 2-related factor 2 (Nrf2), β-catenin, Signal Transducer and Activator of Transcription 3 (STAT3), Activator Protein-1 (AP-1) and Nuclear factor-kappa B (NF-κB)] and pro-inflammatory mediators [such as interleukins (ILs), Tumor Necrosis Factor-α (TNF-α), COX-2 and 5-Lipoxygenase (5-LOX)]. Moreover, polyphenols possess both antioxidant and pro-oxidant properties [112,113]. As a result, polyphenols can influence various processes implicated in carcinogenesis, such as cell cycle progression, apoptosis, autophagy, angiogenesis and invasion [114–117]. For example, curcumin, epigallocatechin gallate (EGCG) and resveratrol have been reported to decrease the proliferation of different cancer cell types [118–120]. Polyphenols have demonstrated promising therapeutic effects also in mesothelioma models. For instance, curcumin has been shown to block autophagy while inducing apoptosis in human mesothelioma cell lines and to significantly increase survival in mice i.p. transplanted with syngeneic mesothelioma cells [121]. Similarly, the flavonoid apigenin and the BH3 mimetic polyphenol (–)-gossypol (AT-101) exhibit significant anti-tumor effects against mesothelioma, both *in vitro* and *in vivo* [122,123]. Recently, interesting findings have also been obtained with the Evening Primrose Extract (EPE) obtained by the plant *Oenothera paradoxa* and containing the polyphenols gallic acid, ellagic acid and catechin. In particular, in mesothelioma cells, EPE treatment resulted not only in the reduction of cell viability and induction of apoptosis, but also in the reversal of Epithelial-to-Mesenchymal Transition (EMT) and inhibition of cell invasion in *in vitro* assays [124,125].

Remarkably, bioactive plant chemicals frequently exert synergistic effects when combined with different anticancer drugs, enhancing tumor suppression while protecting healthy cells, and reducing adverse effects [126,127]. In particular, polyphenols can support both chemotherapy and targeted therapies by modulating multiple cancer pathways, inhibiting tumor growth, and improving drug bioavailability, and their combination with conventional treatments may help to overcome chemoresistance phenomena [128]. Accordingly, recent research has been increasingly focused on exploring whether the use of bioactive compounds such as polyphenols could enhance the efficacy of other therapeutic agents in mesothelioma. For instance, curcumin, one of the most extensively studied polyphenols, has been found to enhance the efficacy of the pan-ErbB tyrosine kinase inhibitor Afatinib on mesothelioma, amplifying its anti-tumor activity both *in vitro* and in mice engrafted with syngeneic mesothelioma cells. This combination appears to exert its effects by potentiating growth inhibition and enhancing the pro-apoptotic cell response, suggesting that polyphenols could be valuable components in combination therapies targeting specific oncogenic pathways [48].

Different polyphenols have been investigated for their ability to sensitize mesothelioma cells to chemotherapy. Zhang et al. demonstrated the ability of curcumin to inhibit the mTOR signaling pathway, a key regulator of chemoresistance in mesothelioma, further supporting the potential of this polyphenol as an adjuvant to different anticancer treatments [129–131]. Beyond curcumin itself, its synthetic analog EF24 has also shown promising effects as a chemosensitizer. Interestingly, in a study by Onen et al., pretreatment with EF24 followed by cisplatin exposure increased the cytotoxic effects of cisplatin alone on the mesothelioma cell line MSTO-211H, while it decreased the DNA-damaging effects exerted by the platinum compound on nonmalignant mesothelial Met-5A cells [19]. Another natural bioactive compound of interest is resveratrol. Lee et al. demonstrated that the combination of resveratrol and the antimetabolite clofarabine has synergistic antiproliferative effects in mesothelioma, but not in nonmalignant mesothelial cells, through the inhibition of Akt and Sp1, two key regulators of cell survival and proliferation [49]. The same research group investigated the effects of resveratrol in combination with cisplatin in different mesothelioma cell lines and reported that the combination of the two compounds caused a synergistic induction of apoptosis, presumably mediated by oxidative mitochondrial damage [50]. Quercetin is a flavonoid well known for its antioxidant properties. Demiroglu-Zergeroglu et al. showed that treatment with quercetin in combination with cisplatin was more effective than the individual agents in inducing cytotoxicity in SPC212 and SPC111 mesothelioma cell lines, likely acting via the activation of the JNK and p38 stress signaling pathways [51,52]. Martinotti et al. showed that the combination of ascorbate with the green tea polyphenol EGCG and the antimetabolite gemcitabine synergistically deregulates the cell cycle and induces apoptosis in REN mesothelioma cells. Moreover, compared to the individual agents, the triple combination caused a stronger reduction in tumor development and metastasis formation in a mesothelioma mouse xenograft model [53].

Despite the promising results obtained in preclinical studies, the use of polyphenols in combination with chemotherapeutic agents has not been clinically investigated yet in mesothelioma patients. Nonetheless, the therapeutic efficacy of combining chemotherapy with the polyphenol curcumin has been assessed in trials on pancreatic, prostate, breast and colorectal cancers, with evidence of therapeutic benefits [132].

Actually, the clinical translation of polyphenol-based combination therapies faces several important challenges. One of the most critical limitations is the low oral bioavailability of polyphenols, which compromises their therapeutic potential. This issue arises from several factors, including polyphenols’ rapid metabolism in the liver and gastrointestinal tract, poor aqueous solubility, and interactions with the food matrix [133]. For instance, at doses ≤1 g/day resveratrol is well-tolerated in humans, but does not reach the plasma concentration required for anticancer efficacy. Higher doses (e.g., 5 g/day) may approach therapeutic levels but are associated with increased risk of adverse effects [134]. On the other hand, the anticancer effectiveness of resveratrol has been found to depend largely on the bioactivity of its major metabolites, prompting research into synthetic derivatives with improved pharmacokinetic profiles. A similar case is curcumin, whose clinical use remains hampered by poor bioavailability and low water solubility [135]. Multiple strategies are currently under development to overcome these limitations, such as the use of structurally modified derivatives, the administration of metabolites, or advanced formulations like nanoparticle-based delivery systems, which can enhance stability, solubility, and tissue accumulation [134,135]. When evaluating the clinical translation of polyphenol-based combination therapies, a further issue to consider is potential drug interactions. Indeed, several polyphenols modulate drug-metabolizing enzymes (such as cytochrome P450 isoforms) and transporters (e.g., P-glycoprotein), which could impact the pharmacokinetics of co-administered chemotherapeutic agents, altering efficacy or increasing toxicity [136]. In addition, most studies have been conducted in cell lines or animal models that do not fully replicate human disease complexity. Future research should therefore focus on conducting well-designed clinical trials with optimized formulations to determine the true potential of these compounds in the clinical setting and whether the combination of polyphenols and chemotherapeutic agents may actually improve therapeutic outcomes in mesothelioma patients.

## 6 Conclusions

Mesothelioma remains a clinical challenge due to its aggressiveness, late diagnosis, and resistance to conventional treatments. The therapeutic options presently available for mesothelioma have limited efficacy and there is an urgency for developing more effective strategies to treat this disease. To address this challenge, research has increasingly focused on combinatorial treatment approaches. As highlighted in this review, numerous efforts are being made to enhance therapeutic efficacy through combined approaches integrating chemotherapy, targeted therapies, vaccines, and natural bioactive compounds like polyphenols.

As a matter of fact, preclinical studies and clinical trials exploring novel combination strategies remain vital for improving the outcomes of patients with mesothelioma. Although some clinical trials have already shown encouraging results, many remain in early phases or are limited by small sample sizes. Thus, further studies are essential to validate these findings and translate them into standardized treatment protocols. The evidence obtained so far supports the continuation of research in this direction in order to develop more effective and personalized therapeutic strategies with the potential to improve patient outcomes in this rare and aggressive tumor.

## Data Availability

Not applicable.
